# Outer Membrane Permeabilization Is an Essential Step in the Killing of Gram-Negative Bacteria by the Lectin RegIIIβ

**DOI:** 10.1371/journal.pone.0069901

**Published:** 2013-07-29

**Authors:** Tsuyoshi Miki, Wolf-Dietrich Hardt

**Affiliations:** The Institute of Microbiology, Department of Biology, ETH Zürich, Zürich, Switzerland; Queen’s University Belfast, United Kingdom

## Abstract

The C-type lectin RegIIIβ can kill certain Gram-positive and Gram-negative bacteria. The susceptibility of *S*. Typhimurium depends on the bacterial growth phase, i.e., bacteria from the logarithmic growth phase do bind RegIIIβ and are subsequently killed. Lipid A is one of the bacterial targets for RegIIIβ. However, at the molecular level, it is not understood how RegIIIβ interacts with and kills Gram-negative bacteria. Here, we show that RegIIIβ interacts with Gram-negative bacteria in two distinct steps. Initially, it binds to surface-exposed lipid A. The lipid A can be shielded by the O-antigen of lipopolysaccharide (LPS), as indicated by the exquisite susceptibility of *wbaP* mutants to RegIIIβ-mediated killing. Increased cell viability after incubation with an anti-lipid A antibody also supports this conclusion. This RegIIIβ-binding permeabilizes the outer membrane to hydrophobic dyes like Ethidium bromide or to bulky bacteriolytic enzymes like lysozyme. Conversely, compromising the outer membrane integrity by the mild detergent Triton X-100 enhances the antibacterial effect of RegIIIβ. Based on our observations, we conclude that RegIIIβ interacts with Gram-negative bacteria in two subsequent steps. Initially, it binds to the outer membrane thus leading to outer membrane permeabilization. This initial step is necessary for RegIIIβ to reach a second, still not well understood target site (presumably localized in the periplasm or the cytoplasmic membrane), thereby triggering bacterial death. This provides novel insights into the outer membrane-step of the bactericidal mechanism of RegIIIβ.

## Introduction

The surface of the mammalian intestine interacts directly with the external environment. Hence, these tissues are continuously exposed to bacteria, viruses, fungi and parasites that could act as pathogens. Epithelial antimicrobial proteins such as α-defensins are of critical importance for keeping the large numbers of microorganisms in check [1]–[3]. In addition to limiting microbial challenges, antimicrobial proteins also affect the composition of gut microbiota and may prevent opportunistic invasion by symbiotic bacteria [2], [4].

The RegIII (regenerating gene family protein III) lectins belong to the epithelial antimicrobial proteins. RegIIIβ and RegIIIγ are expressed in the murine intestine, and have bactericidal activity [5]–[8]. The production of RegIIIβ and RegIIIγ is dramatically increased in response to bacterial colonization and pathogenic infection leading to inflammation [5], [6], [8]. The matured RegIII lectins (molecular mass of ∼16 kDa) are secreted from Paneth cells and epithelial cells of the intestine into the gut lumen [5], [6], and RegIIIβ and RegIIIγ have been shown to protect against pathogens and contribute to the maintenance of microbiota homeostasis [5], [8]–[13]. However, their antimicrobial mechanism is still not completely understood.

RegIIIβ and RegIIIγ have distinct spectra of antimicrobial activity. RegIIIγ can kill Gram-positive bacteria but not Gram-negative bacteria whereas RegIIIβ displays bactericidal activity against both certain Gram-positive and Gram-negative bacteria [5], [8]. For example, RegIIIβ was found to kill various bacteria taken from stationary phase cultures, i.e. *Clostridium butyricum*, *Lactobacillus reuteri*, and different *Escherichia coli* strains but not *Enterococcus faecalis*, *Lactobacillus murinus*, and *Salmonella* Typhimurium [8]. The bactericidal effect of RegIII lectins depends on their ability to bind to target bacterial structures [14], [15]. RegIIIγ binds to Gram-positive bacteria by recognizing peptidoglycan, and then seems to kill by damaging the cell wall and provoking cytoplasmic leakage [5], [14]. Similarly, it is conceivable that RegIIIβ kills Gram-positive bacteria by the same mechanism as RegIIIγ because RegIIIβ can also bind to peptidoglycan [14], [15]. In contrast to RegIIIγ, RegIIIβ can also bind to Gram-negative bacteria. This has been studied best using the Gram-negative enteropathogen *Salmonella* Typhimurium (*S*. Typhimurium). Here, RegIIIβ was shown to bind to the bacterial surface and inhibition studies indicated that it can bind directly to the lipid A moiety of lipopolysaccharide [15]. However, the killing mechanism of RegIIIβ is not well understood and it had remained unclear, how it is linked to lipid A binding.

Interestingly, RegIIIβ was able to kill *S*. Typhimurium taken from a logarithmic growth phase culture. In fact, the bactericidal activity of RegIIIβ against *S*. Typhimurium was found to be strictly dependent on the pathogen’s growth phase and kills *S*. Typhimurium grown at the logarithmic growth phase, but not bacteria taken from stationary cultures [15]. A tripeptide ERN (E134, R135 and N136) and D142 in the Loop 2 of RegIIIβ are involved in bacterial recognition and bactericidal activity [15]. However, the mechanism determining this growth phase dependency had remained enigmatic.

Here, we have analyzed the bactericidal mechanism of RegIIIβ against *S*. Typhimurium. In particular, we focused on the initial interaction with the outer membrane. This demonstrated that binding of surface-exposed lipid A and subsequent outer membrane permeabilization represents an important step of the bactericidal mechanism. Our study provides novel insights into the bactericidal mechanism of RegIII family lectins against Gram-negative bacteria.

## Materials and Methods

### Bacterial Strains

*S*. Typhimurium wild-type strain SL1344 was used in this study [16]. SKI12 harboring a mutation of *wbaP* gene is a SL1344 derivative lacking an LPS O-antigen [17]. Bacteria were diluted (1∶200) from an overnight culture in 20 ml of LB broth (100-ml Erlenmeyer flask) and grown to the indicated OD_600_ under mild aeration (160 rpm) at 37°C. Streptomycin (50 µg/ml) and kanamycin (50 µg/ml) were used when required.

### Antibiotics and Antibodies

Polymyxin B was purchased from Novo Nordisk (Copenhagen, Denmark). Gentamicin and ampicillin were purchased from Axonlab (Baden, Switzerland). Ciprofloxacin was purchased from Bayer (Leverkusen, Germany). Anti-lipid A antibody [26-5] and anti-*Salmonella* Typhimurium LPS antibody [1E6] were purchased from abcam (Cambridge, UK). IgG from mouse serum was purchased from Sigma-Aldrich (St. Louis, MO, USA).

### Purification of Recombinant Proteins

Recombinant untagged-RegIIIβ and its point-mutated variants were prepared as described previously [8], [15]. Briefly, *E*. *coli* expressing RegIIIβ were lysed by sonication, and then the resulting inclusion bodies including RegIIIβ were purified. The purified RegIIIβ inclusion bodies were solubilized in denaturing buffer containing guanidine-HCl and then subjected to refolding buffer containing arginine-HCl to refold the RegIIIβ protein. Finally, the refolded RegIIIβ was purified by dialysis in binding buffer (25 mM 4-morpholineethanesulfonic acid (MES) pH 6.0, 25 mM NaCl).

### *In vitro* Killing Assay

The *in vitro* killing assay was performed as described previously [15]. In brief, bacteria grown at the indicated growth phase were washed and resuspended in binding buffer (25 mM MES pH 6.0, 25 mM NaCl) at a density of 1–3×10^6^ cfu/ml. The diluted bacterial suspension was exposed to RegIIIβ (2.5 or 10 µM) or polymyxin B (1 µg/ml) at 37°C for 30 min. Bacteria were then plated on selective LB media. The recovered cfu were normalized for the original cfu of the inoculum, thus yielding the bacterial “survival” (in %). Preincubation steps using anti-lipid A or mouse IgG were performed for 10 min at 37°C, as indicated.

### Bacterial Binding Assay

The bacterial binding assay was performed as described previously [15]. In brief, 50 µl aliquots (1.0×10^7^ cfu) of *S*. Typhimurium cells grown up to the logarithmic growth phase were pelleted by centrifugation, washed with binding buffer (25 mM MES pH 6.0, 25 mM NaCl), suspended in binding buffer and incubated with various concentrations of anti-lipid A antibody or mouse IgG, as indicated. Ten micrograms of RegIIIβ or BSA (in 50 µl total volume) was added and incubated for 15 min at 37°C. The reaction mixtures were centrifuged at 6000 *g* for 5 min, and the resulting supernatant was isolated, mixed with SDS-PAGE sample buffer and boiled for 5 min. The pellet was washed once with binding buffer and boiled for 5 min in SDS-PAGE sample buffer (50 mM Tris-HCl, pH 6.8, 2% (w/v) SDS, 0.1% (w/v) bromophenol blue, 10% (v/v) glycerol, 100 mM 2-mercaptoethanol). The samples were subjected to SDS-PAGE and analyzed by Coomassie brilliant blue (CBB) staining. The corresponding band of RegIIIβ was identified by comparing with a reaction mixture control containing *S*. Typhimurium, but not RegIIIβ, or purified RegIIIβ proteins.

### Bacterial Whole-cell ELISA

A 96-well microtiter plate (Nunc MaxiSorp® flat-bottom 96 well plate) was coated with 100 µl of *S*. Typhimurium (grown to mid-logarithmic growth phase or stationary phase) in PBS (5×10^7^ cfu/well) for 1 h at 37°C, and incubated at 4°C overnight. After washing with wash buffer (0.05% Tween-20 in dH_2_O), 100 µl of anti-lipid A antibody (0.02 mg/ml) or anti-*Salmonella* Typhimurium LPS antibody (1 µg/ml) in blocking buffer (4% BSA, 0.05% Tween-20 in PBS) were incubated for 1 h at 37°C. After incubation, the wells were washed with washing buffer extensively, 100 µl of anti-mouse IgG conjugated to horseradish peroxidase (HRP, Sigma; 1∶100 for lipid A or 1: 1000 for LPS, diluted in blocking buffer) was incubated for 1 h at 37°C. After extensively washing with wash buffer, ABTS (2,2′-Azino-bis(3-ethylbenzthiazoline-6-sulfonic acid); Calbiochem, San Diego, CA, USA) was added, and then incubated for 15 min at room temperature. The bound HRP was detected by measuring OD_405_ of each well using a Spectra max plus microplate spectrophotometer (Molecular devices, Sunnyvale, CA, USA).

### Outer Membrane Permeability Assay (EtBr Influx Assay)

*S*. Typhimurium grown to the mid-logarithmic growth phase was washed with binding buffer (25 mM MES pH 6.0, 25 mM NaCl), and diluted to OD_600_ = 0.4/ml in binding buffer. The bacterial suspension was incubated with RegIIIβ (2.5 or 5 or 10 µM final concentration) or point-mutant RegIIIβ variants (10 µM), polymyxin B (1 µg/ml), gentamicin (100 µg/ml), ciprofloxacin (1 µg/ml) or BSA (160 µg/ml) for 20 min at 37°C. If necessary, anti-lipid A antibody (16 µg/ml) or mouse IgG (16 µg/ml) were preincubated for 10 min at 37°C. After addition of ethidium bromide (EtBr) (6 µM), the fluorescence of the EtBr-nucleic acid complex was immediately measured by using Cary Eclipse fluorescence spectrometer (Varian Inc., Walnut Creek, CA, USA) with excitation and emission wavelengths of 545 and 600 nm, respectively. The widths of the slits are 5 and 10 nm, respectively.

### Statistical Analysis

Statistical analysis was performed using the Mann Whitney *U*-test or unpaired Student’s *t*-test. Correlation analysis was performed using Pearson’s correlation coefficient. *P*<0.05 was considered to be statistically significant.

## Results

### Binding of RegIIIβ to Surface-exposed Lipid A is Essential for its Bactericidal Activity

Earlier work has established that RegIIIβ binds to the lipid A moiety of LPS and that this bacterial binding is critical for its bactericidal activity against Gram-negative bacteria [15]. In the unperturbed outer membrane, the lipid A moiety of LPS is mostly shielded by the LPS O-antigen. This raised the question if the accessibility of the lipid A might represent a limiting step in RegIIIβ-mediated killing. Therefore, we investigated whether masking of surface-exposed lipid A by using anti-lipid A antibody can inhibit the bactericidal activity of RegIIIβ. We first evaluated binding-specificity of the anti-lipid A antibody. The anti-lipid A antibody used in this study bound to lipid A moiety of purified LPS and extracted lipid A, but not to any other moieties of the purified LPS, especially the O-antigen (Figure S1A and C). In contrast, the anti-LPS antibody bound to the O-antigen of LPS, but not to the lipid A moiety (Figure S1B and D). Furthermore, lipid A in the fractionated outer membrane, but not any other proteins, was detected by the anti-lipid A antibody (Figure S1E–G). Altogether, these results verify the binding specificity of the anti-lipid A antibody., Therefore, this reagent should be suitable for masking the surface-exposed lipid A when incubated with Gram-negative bacteria.

**Figure 1:**
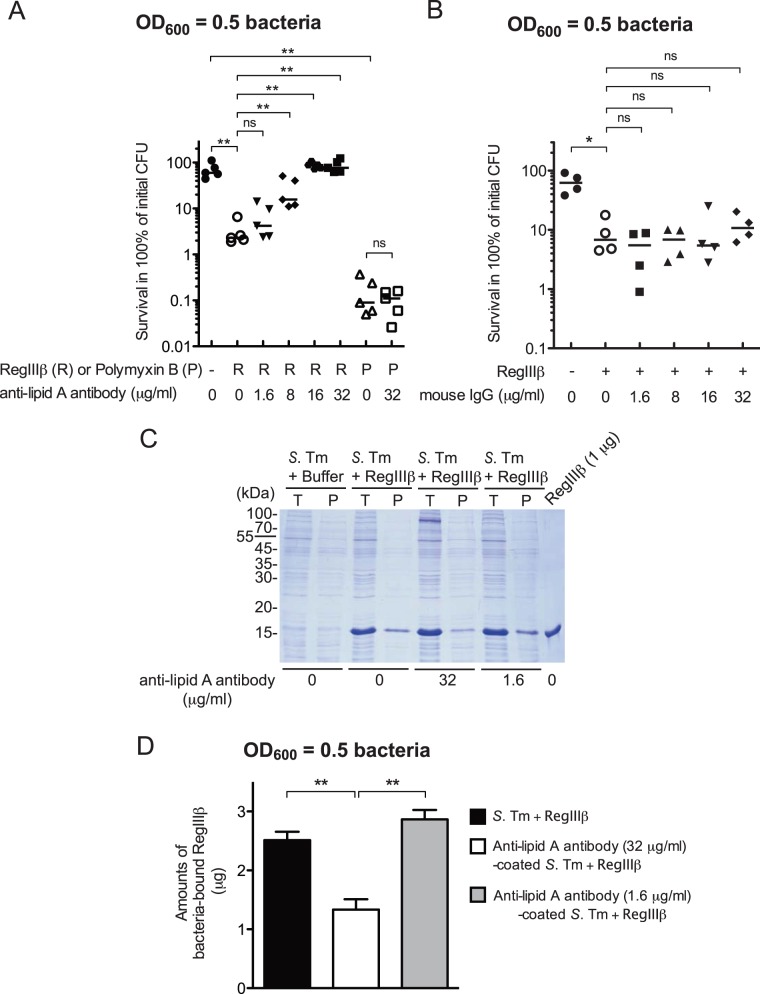
Preincubation with anti-lipid A antibody inhibits the bactericidal effect of RegIIIβ. A) *S*. Typhimurium wild-type (SL1344) from the mid-logarithmic growth phase was incubated with the indicated concentrations of anti-lipid A antibody for 10 min at 37°C. Afterwards, purified RegIIIβ (10 µM) or polymyxin B (1 µg/ml) was added and the mixture was incubated for 30 min at 37°C. Bacterial survival was quantified by dilution-plating. Assays were done in five independent experiments. The bar shows the median. **P<0.01; ns, not significant (Mann-Whitney *U* test). B) *S*. Typhimurium wild-type (SL1344) from the mid-logarithmic growth phase was incubated with the indicated concentrations of mouse IgG for 10 min at 37°C. Purified RegIIIβ (10 µM) was incubated with the preincubated mixture for 30 min at 37°C and bacterial survival was quantified by dilution-plating. Assays were done in four independent experiments. The bar shows the median. *P<0.05; ns, not significant (Mann-Whitney *U* test). C) Bacteria (1×10^7^ cfu) were grown up to the mid-logarithmic growth phase, preincubated with anti-lipid A antibody (1.6 or 32 µg/ml) at 37°C for 10 min, and then incubated with RegIIIβ (10 µg) at 37°C for 15 min (*T*, total fraction). The RegIIIβ bound or unbound to bacteria was separated by centrifugation (*P*, pellet), and then analyzed by SDS-PAGE and CBB staining. D) Quantitative analysis. Amounts of bacteria-bound RegIIIβ (µg) were determined by comparing the band intensity of 1 µg of RegIIIβ as a control. The error bars represent the S.D. of the mean from at least three independent experiments. **P<0.01; ns, not significant (unpaired Student’s *t*-test).

To this end, we preincubated *S*. Typhimurium grown to OD_600_ = 0.5 (note: at OD_600_ = 0.5 the bacteria are sensitive to RegIIIβ-mediated killing; [15]) with an anti-lipid A antibody, before the bacteria were exposed to RegIIIβ. Preincubation with low concentrations (1.6 µg/ml) of anti-lipid A antibody had no detectable effect on the bactericidal activity of RegIIIβ (Fig. 1A). However, at concentrations of ≥8 µg/ml the anti-lipid A antibody inhibited RegIIIβ-mediated killing. Complete inhibition was observed at anti-lipid A antibody concentrations of 16 or 32 μg/ml (Fig. 1A).

It remained unclear if the anti-lipid A antibody specifically inhibited RegIIIβ. Alternatively, it might provide a non-specific shield against LPS-binding agents. To test this, we analyzed the effect of the anti-lipid A antibody on polymyxin B-mediated killing. Polymyxin B is a small cationic antimicrobial antibiotic targeting the lipid A and 3-deoxy-D-*manno*-oct-2-ulosonic acid (Kdo) moieties of LPS [18]. However, unlike RegIIIβ, the bactericidal activity of polymyxin B was not inhibited by the anti-lipid A antibody, even at concentration of 32 μg/ml (Fig. 1A), indicating that the anti-lipid A antibody-mediated masking may not block the PMB-(lipid A)-Kdo interaction. Furthermore, we performed a control with non-specific mouse IgG. Preincubation with the control mouse IgG did not affect the bactericidal activity of RegIIIβ at any antibody concentration (Fig. 1B). This verified that the anti-lipid A antibody specifically inhibited the bactericidal activity of RegIIIβ.

Binding of RegIIIβ to the bacterial surface is necessary for its bactericidal effect [15]. Therefore, we investigated whether the masking surface-exposed lipid A by the anti-lipid A antibody can prevent the binding of RegIIIβ to the bacterial surface. Indeed, preincubation with high concentrations of anti-lipid A antibody (32 μg/ml), but not low concentrations of the same antibody (1.6 μg/ml), inhibited the binding of RegIIIβ to the bacteria (Fig. 1C and 1D). Collectively, these results suggest that the masking surface-exposed lipid A by preincubation with anti-lipid A antibody protects *S*. Typhimurium from the bactericidal effect of RegIIIβ by inhibiting the access of RegIIIβ to lipid A. Moreover, these data indicated that the growing bacteria display “exposed” lipid A moieties on their surface which can be bound by either the anti-lipid A antibody, or by RegIIIβ.

### The Bactericidal Activity of RegIIIβ Correlates with the Amount of Surface-exposed Lipid A

The bactericidal activity of RegIIIβ is dependent on bacterial growth phase, i.e., RegIIIβ kills *S*. Typhimurium cells grown at logarithmic growth phase, but not cells taken from the stationary phase [15]. Together with the data presented in Fig. 1, this suggested that *S*. Typhimurium cells from the logarithmic growth phase display more surface exposed lipid A than cells from the stationary phase. To investigate this, we performed an ELISA assay on whole bacterial cells. To this end, ELISA plates were coated with *S*. Typhimurium grown at mid-log phase (*A*_600_ = 0.5) or the stationary phase (*A*_600_≥1.0) and used to analyze the binding of the anti-lipid A antibody. An antibody directed against the O-antigen served as a positive control, as this epitope should be exposed on logarithmic growth phase and stationary phase bacteria, alike.

**Figure 2:**
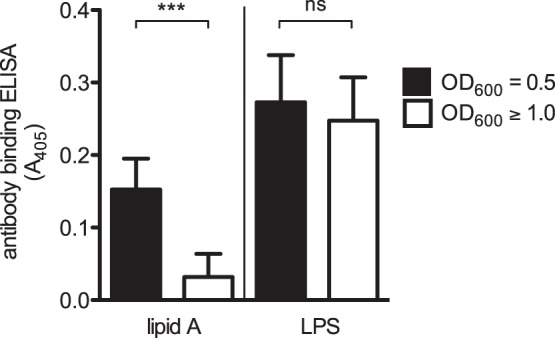
Difference in the amount of surface-exposed lipid A in distinct bacterial growth phases of *S. Typhimurium*. Amounts of exposed lipid A and LPS in *S*. Typhimurium wild-type strain (SL1344) grown at mid-logarithmic growth phase (OD_600_ = 0.5) or at stationary phase (OD_600_≥1.0) were compared by whole-cell ELISA using anti-lipid A or anti-LPS antibodies (Experimental Procedures). The error bars represent the SD of the mean from three independent experiments. ***P<0.001; ns, not significant (unpaired Student’s *t*-test).

*S*. Typhimurium from the mid-log phase (*A*_600_ = 0.5) displayed more surface-exposed lipid A than cells from the stationary phase (*A*_600_≥1.0) (Fig. 2, left side of the figure). In contrast, the anti-O-antigen antibody bound equally well to mid-log and stationary phase bacteria (Fig. 2, right side of the figure). This indicated that the differential binding detected with the anti-lipid A antibody was not attributable to loss or reduced expression of O-antigen. These data provided additional evidence that the high susceptibility of logarithmic growth phase bacteria to RegIIIβ-mediated killing was indeed attributable to increased amounts of surface-exposed lipid A.

### The O-specific Polysaccharide Inhibits the Access of RegIIIβ to Surface-exposed Lipid A

**Figure 3:**
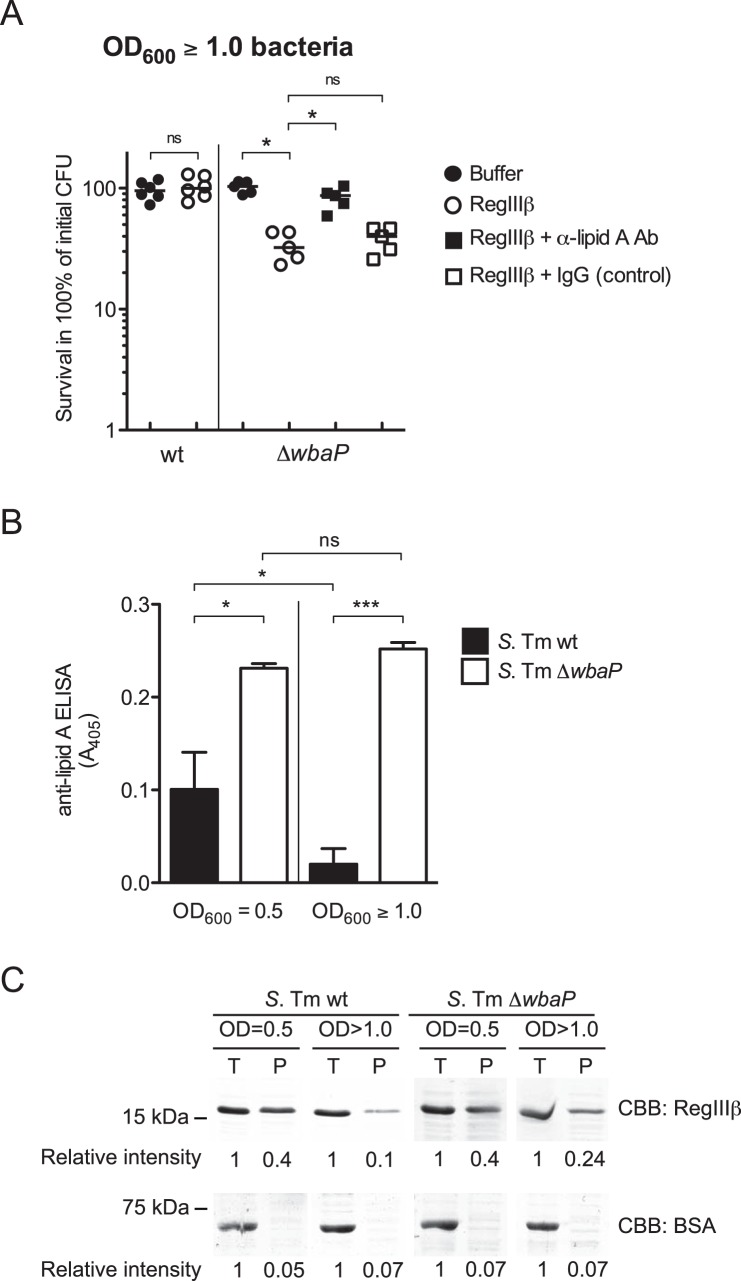
O-antigen deficient *S. Typhimurium* Δ*wbaP* is highly RegIIIβ-susceptible at stationary phase, and displays high amounts of surface-exposed lipid A. A) Inhibition of RegIIIβ killing. *S*. Typhimurium Δ*wbaP* (SKI12) was grown to stationary phase and incubated for 10 min at 37°C with 32 μg per ml of anti-lipid A antibody or mouse IgG. Purified RegIIIβ (10 µM) was incubated with the antibody- or IgG-preincubated SKI12 strain or untreated control bacteria for 30 min at 37°C. Bacterial survival was quantified by dilution-plating. Assays were performed in at least five independent experiments. The bar shows the median. *P<0.05; ns, not significant (Mann-Whitney *U* test). B) Amounts of surface-exposed lipid A in *S*. Typhimurium wild-type strain (*S*. Tm wt) or the O-antigen deficient strain (*S*. Tm Δ*wbaP*) grown at mid-logarithmic growth phase (OD_600_ = 0.5) or at stationary phase (OD_600_≥1.0). Surface binding was analyzed by whole-cell ELISA using the anti-lipid A antibody. The error bars represent the SD of the mean from three independent experiments. *P<0.05; ***P<0.001; ns, not significant (unpaired Student’s *t*-test). C) Bacterial binding assay. Wild type *S*. Typhimurium or the O-antigen deficient strain (*S*. Tm Δ*wbaP*; 1×10^7^ cfu) were grown up to the mid-logarithmic growth phase or the stationary phase, and then incubated with RegIIIβ (10 µg) or BSA (10 µg) at 37°C for 15 min (*T*, total fraction: 50 µl). The RegIIIβ bound or unbound to bacteria was separated by centrifugation (*P*, pellet), and then analyzed by SDS-PAGE and CBB staining. The band intensity was calculated using ImageJ software by defining the relative intensity of the total fraction (*T*) as “1”.

Our previous data have established that the O-specific polysaccharide (O-antigen)-deficient *S*. Typhimurium (Δ*wbaP* mutant) is susceptible to RegIIIβ-mediated killing even when bacteria are taken from the stationary phase [8]. Considering the data presented above, this suggested that the susceptibility of Δ*wbaP* mutant might be explained by the lack of O-antigen mediated shielding of the lipid A. First, this was tested in the antibody shielding assay presented in Fig. 1. Indeed, the bactericidal effect of RegIIIβ against the Δ*wbaP* mutant was inhibited significantly upon preincubation with the anti-lipid A antibody, but not with the control IgG (Fig. 3A). These results confirmed that binding to surface-exposed lipid A is an essential step of RegIIIβ-mediated killing of *S*. Typhimurium Δ*wbaP* taken from the stationary.

In order to substantiate this, we compared the amount of surface-exposed lipid A between wt *S*. Typhimurium and the Δ*wbaP* mutant using the whole-bacterial cell ELISA presented in Fig. 2. Indeed, the Δ*wbaP* mutant displayed significantly higher levels of anti-lipid A antibody-binding than the wt strain. This was true for bacteria from the logarithmic growth phase and from the stationary phase (Fig. 3B). Furthermore, we observed that the *wbaP* mutant bound significantly higher amounts of RegIIIβ, but not BSA as a control, i.e. in the stationary phase, as shown in the bacterial pull-down assay (Fig. 3C). This was in line with our hypothesis that the levels of surface exposed lipid A determine the susceptibility of *S*. Typhimurium to RegIIIβ-mediated killing.

### Compromised Outer Membrane Integrity Increases the Susceptibility to RegIIIβ

**Figure 4:**
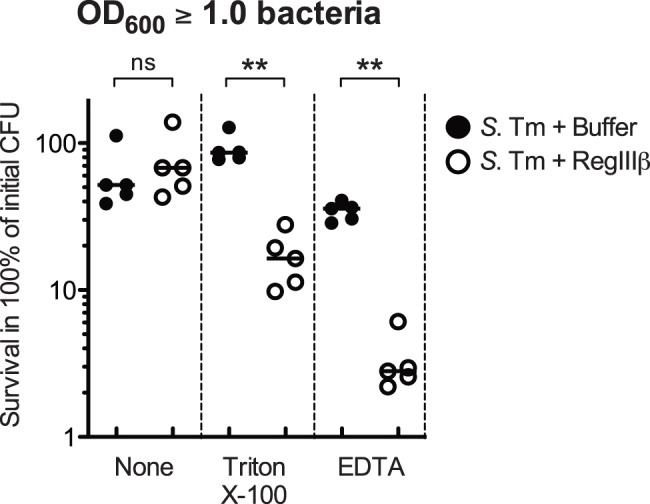
Triton X-100 and EDTA increase the susceptibility to RegIIIβ-killing of *S. Typhimurium* from the stationary phase. The *S*. Typhimurium wild-type strain (*S*. Tm) was grown to stationary phase (OD_600_≥1.0), and coincubated with purified RegIIIβ (10 µM) and Triton X-100 (1%) or EDTA (1 mM) for 30 min at 37°C. Bacterial survival was quantified by dilution-plating. Assays were repeated five times. The bars show the median. ** *P*<0.01; ns, not significant (Mann-Whitney *U* test).

To further assess the critical role of outer membrane structure in RegIIIβ-mediated killing, we investigated whether compromising the outer membrane barrier integrity may increase the susceptibility to RegIIIβ. Control experiments confirmed that *S*. Typhimurium was completely resistant to the killing by RegIIIβ when taken from the stationary phase (Fig. 4). However, treatment with sublethal concentrations of Triton X-100 (a mild detergent) or EDTA (a Mg^2+^ chelator known to disrupt barrier of outer membrane) rendered the stationary phase bacteria highly susceptible to RegIIIβ (Fig. 4). Furthermore, Mg^2+^ does not seem to be essential for RegIIIβ activity, as RegIIIβ displayed a pronounced bactericidal activity in the presence of EDTA.

Collectively, these results suggest that the structure of the LPS defines the degree of susceptibility of *S*. Typhimurium against RegIIIβ-mediated killing. RegIIIβ binding to the lipid A of the outer membrane seems to be an essential step of the killing mechanism.

### RegIIIβ Reduces the Outer Membrane Barrier Function of *S*. Typhimurium

**Figure 5:**
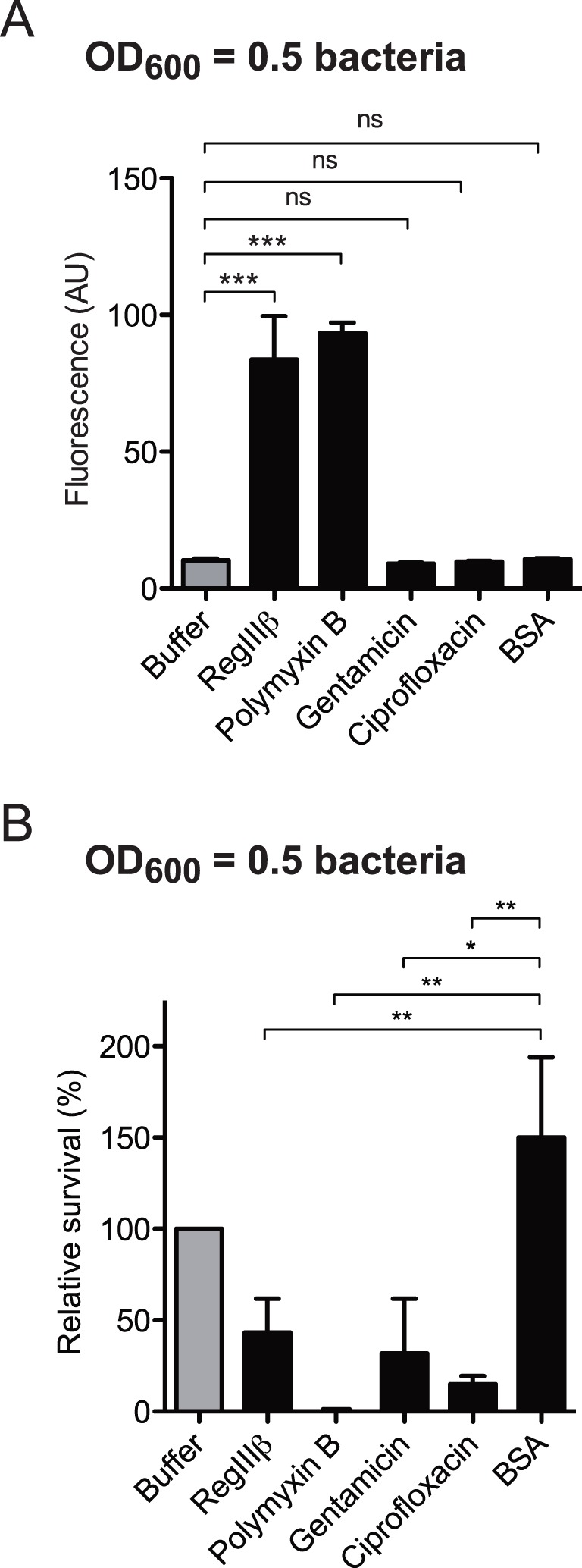
RegIIIβ permeabilizes the outer membrane. A) The outer membrane permeability of wild-type *S*. Typhimurium from the mid-logarithmic growth phase was measured in the presence of RegIIIβ (10 µM), polymyxin B (1 µg/ml), gentamicin (10 µg/ml), ciprofloxacin (1 µg/ml) or BSA (160 µg/ml) using the ethidium bromide influx assay. The error bars represent the SD of the mean from at least three independent experiments. *** *P*<0.001; ns, not significant (unpaired Student’s *t*-test). B) Activity control of the antimicrobial agents. Bacterial survival (%) of bacteria from the assay shown in (A) was assessed by plating. The data was plotted as 100% of the buffer-treated samples. The error bars represent the SD of the mean from at least three independent experiments. ** *P*<0.01; *** *P*<0.001; ns, not significant (unpaired Student’s *t*-test).

First, we were testing if RegIIIβ does indeed affect outer membrane barrier function. To assess outer membrane strength, we performed an ethidium bromide (EtBr) influx assay [19]. EtBr, a DNA-intercalating fluorescent dye, cannot effectively enter *S*. Typhimurium, as long as the outer membrane barrier is intact. However, upon passing the outer membrane, EtBr rapidly traverses the cytoplasmic membrane, enters the bacterial cytosol and binds to intracellular nucleic acids. The latter can be detected by fluorescence spectrometry, as EtBr displays strongly enhanced fluorescence when intercalating with nucleic acids. We also employed polymyxin B (disrupts the outer membrane [20], [21]), gentamicin and ciprofloxacin two antibiotics known to kill *S.* Typhimurium but would (most likely) leave the outer membrane intact. BSA served as a negative control. Enhanced fluorescence signal intensities were found with RegIIIβ and with polymyxin B, whereas gentamicin-, ciprofloxacin-, or BSA-treated cells did not display enhanced signal intensity (Fig. 5A). Plating assays determining the number of colony forming units verified that RegIIIβ, polymyxin B and the antibiotics displayed the expected antimicrobial activities whereas BSA did not affect bacterial viability (Fig. 5B). Please note that this assay employed 10-fold higher bacterial densities than the other assays employed in our study. This explains why just 50% of the bacteria are indeed killed by RegIIIβ in the control experiment (Fig. 5B). In conclusion, these data established that RegIIIβ does indeed permeabilize the outer membrane of *S*. Typhimurium.

### RegIIIβ-mediated Membrane Permeabilization Requires the Bacterial Binding of RegIIIβ

**Figure 6:**
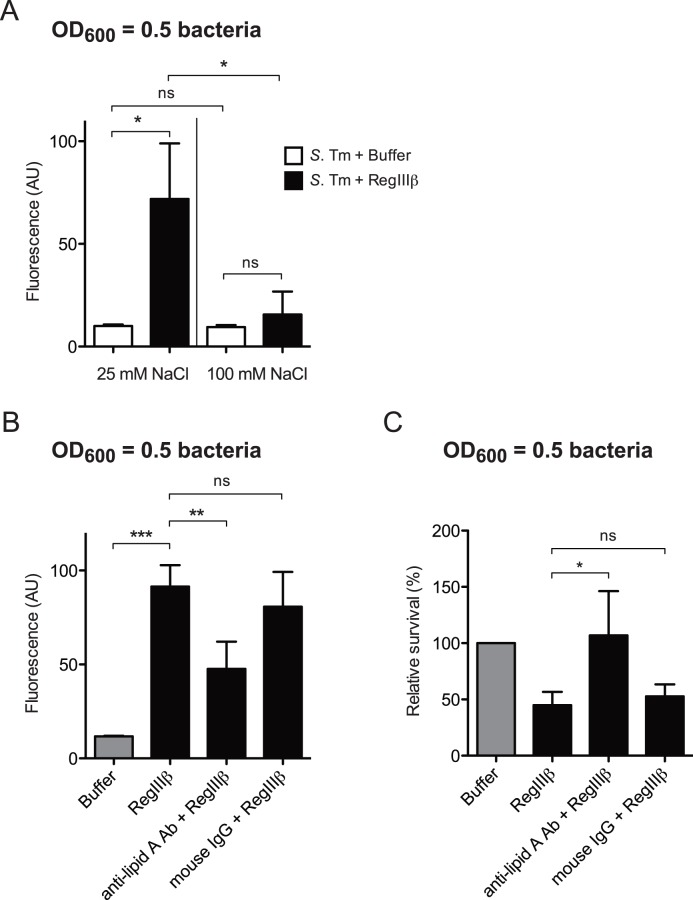
Surface binding is required for RegIIIβ-mediated outer membrane permeabilization. A) Salt-concentration dependency of RegIIIβ-mediated membrane permeabilization. Outer membrane permeabilization in wild-type *S*. Typhimurium (*S*. Tm) from the mid-logarithmic growth phase by RegIIIβ (10 µM) in the presence of 25 mM or 100 mM sodium chloride. The outer membrane permeability was measured usingthe ethidium bromide influx assay. The error bars represent the SD of the mean from at least three independent experiments. * *P*<0.05; ns, not significant (unpaired Student’s *t*-test). B) Preincubation with the anti-lipid A antibody inhibits the RegIIIβ-mediated outer membrane permeabilization. RegIIIβ (10 µM)-mediated outer membrane permeability of wild-type *S*. Typhimurium from the mid-logarithmic growth phase preincubated with anti-lipid A antibody (16 µg/ml) or mouse IgG (16 µg/ml) was analyzed using the ethidium bromide influx assay. The error bars represent the SD of the mean from at least three independent experiments. ** *P*<0.01; *** *P*<0.001; ns, not significant (unpaired Student’s *t*-test). C) Control experiment assessing bacterial survival in the ethidium bromide influx assay (*B*). Relative survival (%) was evaluated as 100% of the buffer-treated samples. The error bars represent the SD of the mean from at least three independent experiments. ** *P*<0.01; *** *P*<0.001; ns, not significant (unpaired Student’s *t*-test).

Our previous data have shown that high concentrations of NaCl (more than 75 mM) completely inhibits binding of and killing by RegIIIβ [15]. Therefore, to investigate whether the bacterial binding is required for membrane permeabilization by RegIIIβ, we performed the EtBr influx assay in the presence of 100 mM or 25 mM NaCl. High concentrations of NaCl inhibited the RegIIIβ-mediated membrane permeabilization (Fig. 6A).

Furthermore, we investigated whether RegIIIβ-mediated membrane permeabilization requires the binding to surface-exposed lipid A. Preincubation with the anti-lipid A antibody, but not control IgG, significantly reduced the fluorescence signal intensity (Fig. 6B). Control assays monitoring bacterial viability verified that the anti-lipid A antibody indeed inhibited RegIIIβ-mediated killing under the conditions of this assay (Fig. 6C). In conclusion, the binding of RegIIIβ to surface-exposed lipid A in the outer membrane is essential for outer membrane permeabilization.

### The Ability of RegIIIβ to Permeabilize the Outer Membrane Correlates with the Bactericidal Activity

**Figure 7:**
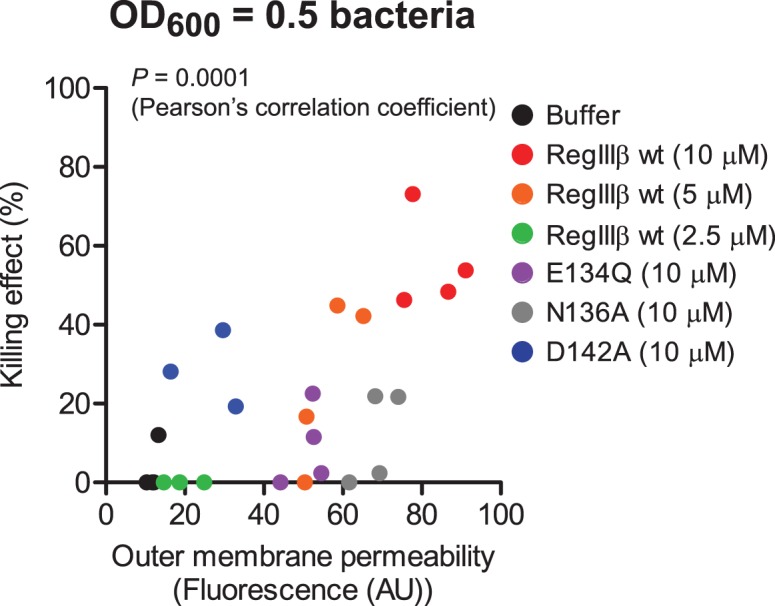
The ability of RegIIIβ to permeabilize outer membrane is correlated with its bactericidal activity. Outer membrane permeability was analyzed using the ethidium bromide influx assay. We analyzed logarithmic growth phase wild-type *S*. Typhimurium incubated with RegIIIβ wt (2.5, or 5, or 10 µM) and the RegIIIβ point mutants E134Q (10 µM), N136A (10 µM) and D142A (10 µM). Killing (%) was analyzed by dilution-plating. The correlation analysis was performed using Pearson’s correlation coefficient (*P* = 0.0001).

Next, we examined whether the ability of RegIIIβ to permeabilize outer membrane correlates with its bactericidal activity. A dose-dependent enhancement of the fluorescence signal intensity was observed upon incubating logarithmic growth phase bacteria with 2.5 µM, 5 µM or 10 µM RegIIIβ (Fig. 7). Interestingly, sublethal concentrations of RegIIIβ (2.5 and 5 µM) were still capable of eliciting detectable outer membrane permeabilization (Fig. 7, green and orange symbols).

The Loop 2 mutants E134Q and N136A of RegIIIβ, which display attenuated bactericidal activity [15], also displayed reduced permeabilization (Fig. 7). The mutant D142A showed a slightly different effect: it had a detectable bactericidal effect already at a concentration yielding just slightly enhanced outer membrane permeability. The slightly different phenotypes of these two RegIIIβ mutants might be explained by different capacities to bind to lipid A [15]. I.e. the more pronounced outer membrane permeability of D142A could be attributable to a reduced capacity to bind lipid A [15]. Additional work would have to confirm the significance of this observation. Altogether, these results indicate that the ability of RegIIIβ to permeabilize the outer membrane correlates with its bactericidal activity (Pearson’s correlation coefficient, *P* = 0.0001). Wild type RegIIIβ seems to permeabilize outer membrane before killing can be detected.

### RegIIIβ-mediated Membrane Permeabilization Sensitizes *S*. Typhimurium to Sublethal Concentrations of Lysozyme or Triton X-100

**Figure 8:**
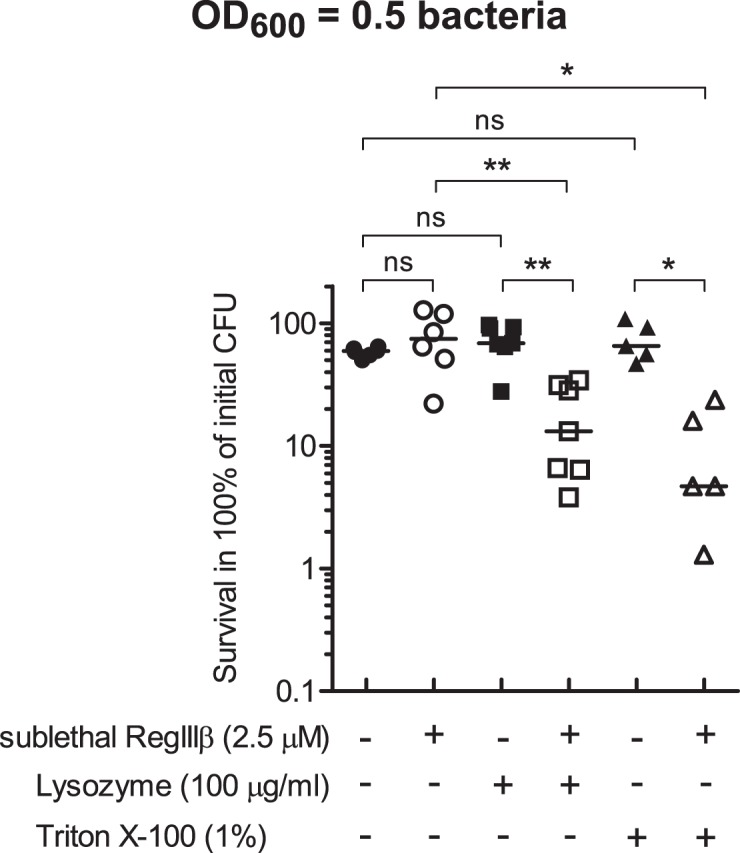
The outer membrane permeabilization by RegIIIβ sensitizes *S. Typhimurium* to lysozyme or Triton X-100. The *in vitro* killing assay was performed using wild-type *S*. Typhimurium from the mid-logarithmic growth phase. Sublethal concentrations of RegIIIβ (2.5 µM), lysozyme (100 µg/ml), Triton X-100 (1%), or the indicated mixtures were incubated with the bacteria for 30 min at 37°C. Bacterial survival was quantified by dilution-plating. Assays were done in at least five independent experiments. The bar shows the median. * *P*<0.05; ns, not significant (Mann-Whitney *U* test).

Effective increase in outer membrane permeability sensitizes Gram-negative bacteria to hydrophobic antibiotics such as erythromycin or bacteriolytic reagents including lysozyme or Triton X-100 that cannot traverse the outer membrane effectively [22], [23]. Therefore, we next investigated whether RegIIIβ sensitizes *S*. Typhimurium to sublethal concentrations of lysozyme or Triton X-100. In logarithmic growth phase *S*. Typhimurium, sublethal concentrations of RegIIIβ (2.5 µM) increase the outer membrane permeability slightly (Fig. 7), but did not (yet) affect bacterial viability (Fig. 7 and 8). One hundred microgram per ml of lysozyme or 1% of Triton X-100 alone also had no effect on bacterial viability (Fig. 8). In contrast, co-incubation with RegIIIβ and lysozyme or Triton X-100 significantly reduced the number of viable bacteria (Fig. 8). These results confirm that sublethal doses of RegIIIβ can permeabilize the outer membrane and that this sensitizes *S*. Typhimurium to detergents like Triton X-100 or bacteriolytic compounds such as lysozyme which interact with periplasmic target sites.

## Discussion

We have previously shown that the bactericidal effect of RegIIIβ depends on the bacterial growth phase. *S*. Typhimurium from the log (not the stationary) phase, was efficiently bound and killed by RegIIIβ [15]. Competitive binding data established that the lipid A moiety of LPS was one target for RegIIIβ. Here, we have extended this work by demonstrating that the binding of RegIIIβ to surface-accessible lipid A is absolutely essential for its bactericidal effect. This surface binding seems to facilitate permeabilization of the outer membrane and represents the initial step of the interaction of RegIIIβ with Gram-negative bacteria.

Surface accessibility of lipid A may also explain the growth phase dependence of the antimicrobial activity of RegIIIβ. In the logarithmic growth phase (but not the stationary phase), *S*. Typhimurium displays high amounts of lipid A on its surface as indicated by its increased binding capacity for anti-lipid A antibody (this work) and for RegIIIβ [15]. Furthermore, a Δ*wbaP* mutant which lacks O-specific polysaccharide, is susceptible to RegIIIβ not only in the logarithmic growth phase but also in the stationary phase. Again, this went along with increased levels of surface-exposed lipid A in both growth phases. Together, these data suggest that the O-side chain provides a steric shield for the lipid A moiety. In wild type *S*. Typhimurium, this shield seems to be less effective in the logarithmic growth phase than in the stationary phase (Figure S2A–B). The Δ*wbaP* mutant lacks this shield and is consequently susceptible to RegIIIβ binding (and killing) in either growth phase (Figure S2C).

Different scenarios are conceivable for explaining why wild type *S*. Typhimurium remains susceptible to RegIIIβ surface binding during the log phase. First of all, the outer leaflet of the outer membrane is composed not only of LPS, but also harbors significant quantities of membrane proteins, including porins. In logarithmic growth phase bacteria, LPS is thought to account for 73.1% of the outer surface of *S*. Typhimurium [24]. Therefore, it is conceivable that RegIIIβ could get access to lipid A by passing through the remaining area (26.9%) (Figure S2A). Alternatively, the surface-exposed lipid A might be located at specific sites like the growth zones of the cell wall or the septum at division sites (Figure S2A). These sites are clearly more prominent in the logarithmic growth phase than in the stationary phase. It will be an interesting topic for future work to assess these hypotheses.

In this study, we showed that when stationary phase bacteria (which are normally resistant to RegIIIβ) were treated with agents compromising outer membrane integrity, i.e. Triton X-100, *S*. Typhimurium becomes highly susceptible to RegIIIβ-mediated killing. Moreover, Triton X-100 was able to reduce the bactericidal doses of RegIIIβ which are required to kill *S*. Typhimurium from the log growth phase. Furthermore, this allowed outer membrane passage of lysozyme (14.3 kDa), a small protein of similar size as RegIIIβ (16 kDa), which can kill Gram-negative bacteria by enzymatic digestion of the murein sacculus, a structure located within the periplasm. These observations indicated that permeabilizing the outer membrane by RegIIIβ is an important first step of the bactericidal mechanism of this antimicrobial lectin against wild type *S*. Typhimurium.

It is still unclear how RegIIIβ triggers bacterial death upon traversal of the outer membrane. The substantial (though reduced) bactericidal dose of RegIIIβ required to kill Triton X-100 treated bacteria, suggests that this second step requires significant amounts of RegIIIβ. Our data suggest that this RegIIIβ must traverse the outer membrane, presumably to reach the bactericidal target site somewhere in the periplasm or the cytoplasmic membrane. In the case of RegIIIβ, these targets are still unknown. Recently, it has been shown that peptidoglycan recognition proteins (PGRPs) kill Gram-positive and Gram-negative bacteria by activating the stress-control two-component regulatory system (CssR-CssS for *Bacillus subtilis* or CpxR-CpxA for *Escherichia coli*), the accompanying hydroxyl radical production and by membrane depolarization, but not membrane permeabilization [25]. It is tempting to speculate that RegIIIγ, which can bind peptidoglycan of Gram-positive bacteria as well as PGRPs, has the same killing mechanism as PGRPs. However, it remains to be elucidated whether this holds true for RegIIIβ-mediated killing of *S*. Typhimurium.

In conclusion, the bactericidal lectin RegIIIβ seems to kill Gram-negative bacteria by a mechanism involving (at least) two distinct steps. In the first step, RegIIIβ binds to surface-exposed lipid A and permeabilizes the outer membrane. The O-antigen confers some protection against this binding-step. The second, bactericidal step seems to be distinct from the initial outer membrane-interaction and awaits further analysis. Our findings advance the mechanistic understanding of the antimicrobial activity of RegIII-familiy lectins and prepare the ground for analysis of the second, bactericidal step.

## Supporting Information

Figure S1 (EPS)

Figure S2 (EPS)

File S1 (DOCX)
