# Supplementary material for: Outer Membrane Permeabilization Is an Essential Step in the Killing of Gram-Negative Bacteria by the Lectin RegIIIβ
Source: PLoS One. 2013 Jul 29;8(7):e69901. doi: 10.1371/journal.pone.0069901 (PMC3726741; doi:10.1371/journal.pone.0069901)
Supplement: File S1 [file pone.0069901.s003.docx]

**MS ID: PONE-D-13-19373 revised v3-1**

**SUPPORTING INFORMATION**

**Full title:**

**Outer membrane permeabilization is an essential step in the killing of Gram-negative bacteria by the Lectin RegIIIβ**

**Short title:**

*Bactericidal mechanism of RegIIIβ*

**Authors and Affiliations:**

Tsuyoshi Miki^1*^ & Wolf-Dietrich Hardt^1*^

^1^ the Institute of Microbiology, Department of Biology, ETH Zürich, 8093 Zürich, Switzerland.

^*^ To whom correspondence should be addressed: Tsuyoshi Miki, Institute of Microbiology, ETH Zürich; current address: Department of Microbiology, School of Pharmacy, Kitasato University, 5-9-1 Shirokane, Minato-ku, 108-8641 Tokyo, Japan, Tel.: +81-3-5791-6256; Fax; +81-3-3444-4831; E-mail: [mikit@pharm.kitasato-u.ac.jp](mailto:mikit@pharm.kitasato-u.ac.jp) and Wolf-Dietrich Hardt, Institute of Microbiology, ETH Zürich, Wolfgang-Pauli-Strasse 10, CH-8093 Zürich, Switzerland, Tel.: +41-44-632-5143; Fax; +41-44-632-1129; E-mail: [hardt@micro.biol.ethz.ch](mailto:hardt@micro.biol.ethz.ch)

**SUPPORTING PROTOCOLS**

**Bacterial fractionation**

*S*. Typhimurium wild-type (SL1344) from the mid-logarithmic growth phase was fractionated into periplasmic, cytoplasmic, inner membrane, and outer membrane fractions as described previously [[1](#_ENREF_1)]. The protein concentrations of fractionated samples were determined by using the bicinchoninic acid (BCA) protein assay (Pierce, Rockford, USA). For verifying subcellular fractionation, samples were subjected to 12% Glycine-SDS-PAGE gel electrophoresis and transferred to polyvinylidene difluoride membranes (Immobilon, Millipore; Billerica, MA, USA). Then, we performed an immunoblot analysis using anti-DnaK (cytoplasmic) and anti-outer membrane proteins (anti-OMPs [[2](#_ENREF_2)]; outer membrane; kind gift from Jörg Vogel) antibodies.

**Reagents and antibodies**

Pure LPS was prepared as described previously [[3](#_ENREF_3)]. Lipid A was purchased Sigma-Aldrich (St. Louis, MO, USA). Anti-DnaK antibody was purchased from abcam (Cambridge, UK). Anti-OMPs antibody was provided by Jörg Vogel (University of Würzburg). Anti-mouse IgG conjugated to alkaline phosphatase and anti-rabbit IgG conjugated to alkaline phosphatase were purchased from Sigma-Aldrich.

**SDS-PAGE and Western blot analysis**

The samples were subjected to 16.5% Tricine-SDS-PAGE gel or 12% Glycine-SDS-PAGE gel, and transferred to PVDF membranes (Immobilon, Millipore) for immunoblotting. As a detectable antibody, anti-mouse IgG conjugated to alkaline phosphatase or anti-rabbit IgG conjugated to alkaline phosphatase was used. The activity of an alkaline phosphatase was visualized using SIGMA*FAST* BCIP/NBT (Sigma).

**SUPPORTING FIGURE LEGENDS**

**FIGURE S1.** **Binding specificity of anti-lipid A antibody.** A-D) Purified LPS [[3](#_ENREF_3)] and extracted lipid A were subjected to SDS-PAGE with 16.5% Tricine-SDS-PAGE gel or 12% Glycine-SDS-PAGE gel. Western blot analysis was done by using anti-lipid A antibody (*A* and *C*) or anti-LPS antibody (*B* and *D*). E) Bacterial fractionation. *S*. Typhimurium wild-type (SL1344) from the mid-logarithmic growth phase was fractionated as described previously [[1](#_ENREF_1)]. Fractionated samples (0.5 µg of periplasmic fraction, or 10 µg of each other samples) were analyzed to 12% Glycine-SDS-PAGE and Western blotting using anti-DnaK and anti-OMPs antibodies. *P*, periplasmic fraction; *C*, cytoplasmic fraction; *I*, inner membrane fraction; *O*, outer membrane fraction. F) and G) The fractionated samples were resolved on 16.5% Tricine- or 12% Glycine-SDS-PAGE and analyzed by Western blotting using anti-lipid A antibody. *P*, periplasmic fraction; *C*, cytoplasmic fraction; *I*, inner membrane fraction; *O*, outer membrane fraction.

**FIGURE S2. Model for Gram-negative bacterial recognition of RegIIIβ.** A) Recognition for *S*. Typhimurium wild-type strain from the mid-logarithmic growth phase. Bacteria are frequently dividing during this phase. O-antigen of LPS prevents from the access of RegIIIβ to lipid A. RegIIIβ bypasses this blockage by passing LPS-less area where porins or other outer membrane proteins are present. Alternatively, the regular arrangement of LPS molecules might be compromised at sites of membrane growth and/or division-septum formation. Here, RegIIIβ could get access to lipid A. B) Recognition for *S*. Typhimurium wild-type strain from the stationary phase. Almost all bacteria are growing slowly and are not dividing. RegIIIβ cannot bind to lipid A effectively by the O-antigen-mediated shielding. C) *S*. Typhimurium *wbaP* mutant lacking O-antigen of LPS. RegIIIβ can bind to lipid A effectively even when bacteria are grown in the stationary phase.

**SUPPORTING REFERENCES**

1. Miki T, Shibagaki Y, Danbara H, Okada N (2009) Functional characterization of SsaE, a novel chaperone protein of the type III secretion system encoded by *Salmonella* pathogenicity island 2. J Bacteriol 191: 6843-6854.

2. Papenfort K, Bouvier M, Mika F, Sharma CM, Vogel J (2010) Evidence for an autonomous 5' target recognition domain in an Hfq-associated small RNA. Proc Natl Acad Sci U S A 107: 20435-20440.

3. De Castro C, Parrilli M, Holst O, Molinaro A (2010) Microbe-associated molecular patterns in innate immunity: Extraction and chemical analysis of gram-negative bacterial lipopolysaccharides. Methods Enzymol 480: 89-115.
